# A hundred years of *Dunaliella *research: 1905–2005

**DOI:** 10.1186/1746-1448-1-2

**Published:** 2005-07-04

**Authors:** Aharon Oren

**Affiliations:** 1 The Institute of Life Sciences, The Hebrew University of Jerusalem, 91904 Jerusalem, Israel; 2 the Moshe Shilo Minerva Center for Marine Biogeochemistry, The Hebrew University of Jerusalem, 91904 Jerusalem, Israel

## Abstract

A hundred years have passed since the description of the genus *Dunaliella*, the unicellular green alga which is responsible for most of the primary production in hypersaline environments worldwide. The present paper provides an historical survey of research on *Dunaliella*, from the early work in the 19^th ^century to the thorough taxonomic studies by Teodoresco, Hamburger, Lerche and others from the beginnig of the 20^th ^century onwards. It attempts to trace the origin of some of the most important breakthroughs that have contributed to our present understanding of this alga that plays such a key role in many hypersaline environments.

## 1. Introduction

A hundred years have passed since the description of the genus *Dunaliella*, the unicellular green alga which is responsible for most of the primary production in hypersaline environments worldwide. First sighted in 1838 in saltern evaporation ponds in the south of France by Michel Felix Dunal [1], it was named after its discoverer by Teodoresco in 1905 [2].

In the century that has elapsed since its formal description, *Dunaliella *has become a convenient model organism for the study of salt adaptation in algae. The establishment of the concept of organic compatible solutes to provide osmotic balance was largely based on the study of *Dunaliella *species. Moreover, the massive accumulation of *β*-carotene by some strains under suitable growth conditions has led to interesting biotechnological applications.

The present paper provides an historical survey of research on *Dunaliella*, from the early work in the 19^th ^century to the thorough taxonomic studies by Teodoresco, Hamburger, Lerche and others from the beginning of the 20^th ^century onwards. It attempts to trace – often through quotations from the original articles – the origin of some of the most important breakthroughs that have contributed to our present understanding of this alga that plays such a key role in many hypersaline environments.

Extensive additional information on the alga can be found in a review by Ginzburg [3], in the multi-author review edited by Avron and Ben-Amotz [4], and in my monograph on halophilic microorganisms and their environments [5].

## 2. Reports on *Dunaliella *Prior to 1905

**Figure 1:**
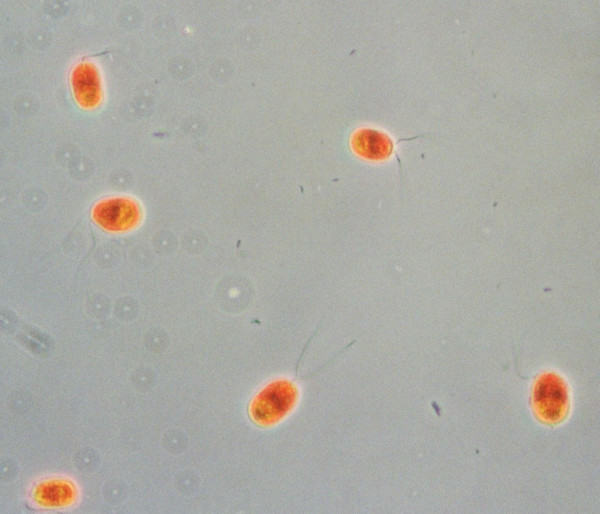
*Dunaliella salina *cells from the crystallizer brine of the salterns in Eilat, at the Red Sea coast of Israel.

The first description of a unicellular biflagellate red-colored alga living in concentrated brines (Fig. 1) was given in 1838 by Dunal [1], who reported occurrence of the organism we know today as *Dunaliella salina *in the salterns of Montpellier, on the Mediterranean coast of France. He named the organisms observed *Haematococcus salinus *and *Protococcus*. The discovery of these algae was made in the framework of an investigation, invited by the Académie des Sciences, Paris, of the cause of the red coloration of saltern brines. At the time it was widely assumed that chemical and physical parameters are responsible for the coloration of these brines. Dunal refuted an earlier claim that the color is due to the brine shrimp *Artemia salina*. The Académie then appointed a committee to reexamine the matter, and this committee confirmed Dunal's finding [6]; see also [7]. Another idea brought forward during that period was that *Artemia *contributes to the color due to the partially digested and decaying red flagellates ("*Monas Dunalii*") present in its intestine [8]. Nowadays it is clear that, although *β*-carotene-rich *Dunaliella *salina are indeed present in the saltern ponds, most of the coloration of the crystallizer brine is caused not by the algae but by red halophilic Archaea instead [9,10].

In the course of the 19^th ^century, Dunal's red flagellate algae has been observed by other biologists as well in salt lakes and other hypersaline sites in Crimea [11,12], Algeria [13], Lorraine, France [14] and Romania [15]. Different names were attached to the organism by each investigator [1,8,11,13,15-20] (Table 1).

**Table 1:** Names attached to the red-colored unicellular flagellate algae observed in hypersaline brines, 1838–1906.

Name	Author
*Haematococcus salinus*	Dunal [1]
*Protococcus salinus*	Dunal [1]; Geleznow [11]
*Monas Dunalii*	Joly [8]; Blanchard [16]; Butschinsky [12]
*Diselmis Dunalii*	Dujardin [17]
*Chlamydomonas Dunalii*	Cohn [18]; Blanchard [13]; Bujor [15]
*Sphaerella lacustris *var. *Dunalii*	Hansgirg [19]
*Dunaliella salina*	Teodoresco [2,20]

## 3. The Description of the Genus *Dunaliella*

The year 1905 saw the publication of two papers presenting in-depth descriptions of *Dunaliella *as a new genus, one by E.C. Teodoresco from Bucharest [2] and the second written by Clara Hamburger from Heidelberg [7]. Teodoresco's publication preceded that by Hamburger, who only learned about the Teodoresco paper when finalizing the writing of her own article [7]:

"Anfang März wollte ich an die Ausarbeitung meiner Notizen gehen, als ich am 10. März von Herrn Prof. Lauterborn eine Arbeit von Teodoresco mit dem Titel: "Organisation et développement du *Dunaliella*, nouveau genre de Volvocaceae – Polyblepharidée erhielt, welche as Separatdruck aus dem botanischen Centralblatt soeben versendet war. *Dunaliella *ist der von mir untersuchte Organismus, den ich schon als Vertreter einer neuen Gattung erkannt hatte. Unsere Resultaten stimmten in vielen Punkten überein, in anderen müssen meiner Ansicht nach erst weitere Untersuchungen die entgültige Entscheidung bringen. Da jedoch meine Studien, besonders bezüglich des innern Baues eingehender sind (Teodoresco hat nur lebendes Material untersucht) und ich auch einige noch bestehende Lücken ausfüllen kann; da ferner alle meine Resultate unbeinflußt von denen Teodoresco's erhalten wurden, so möchte ich sie dennoch veröffentlichen."

[In the beginning of March [1905] I wanted to start to work out my notes, when on March 10 I received from Prof. Lauterborn a paper by Teodoresco entitled: "Organization and development of *Dunaliella*, a new genus of the Volvocida – Polyblepharidae", which was just sent as offprint from the Botanisches Centralblatt. *Dunaliella *is the organism that I had been investigating, and that I had already recognized as representative of a new genus. Our results corresponded in many respects, while in other respects I am of the opinion that further investigations will have to decide. However, because my studies, especially with respect to the internal structure, are more thorough (Teodoresco had studied only live material) and I also can fill in certain still existing gaps in the knowledge, and also because my results were obtained independently of those of Teodoresco, I still would like to publish them.]

**Figure 2:**
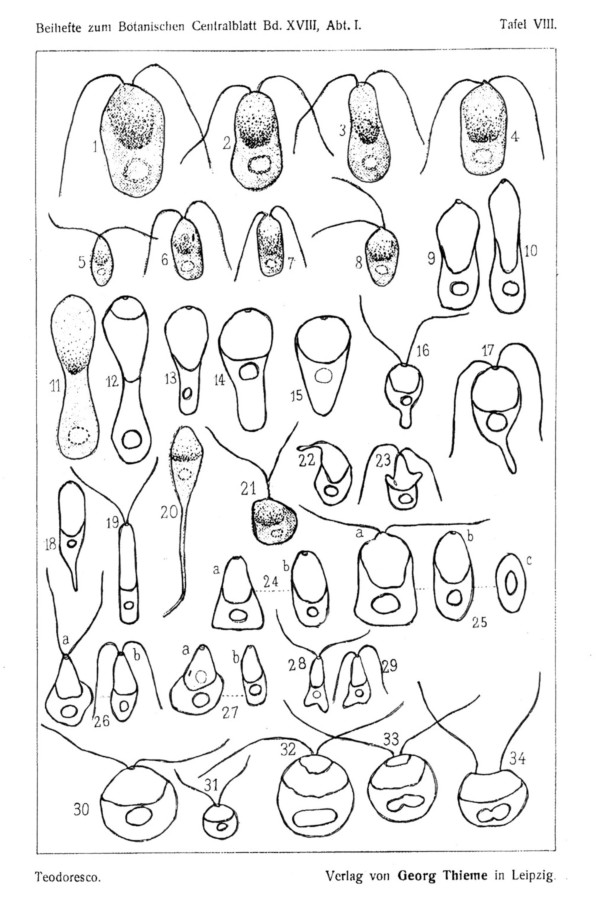
Drawings by Hamburger (1905) of red cells (*Dunaliella salina*) (1–4) and green cells (*D. viridis*) (5–8), diverse shapes observed in a drop that becomes more concentrated by evaporation (9–29), spherical forms obtained upon dilution (30–31), and initiation of cell division (32–34). From [2].

**Figure 3:**
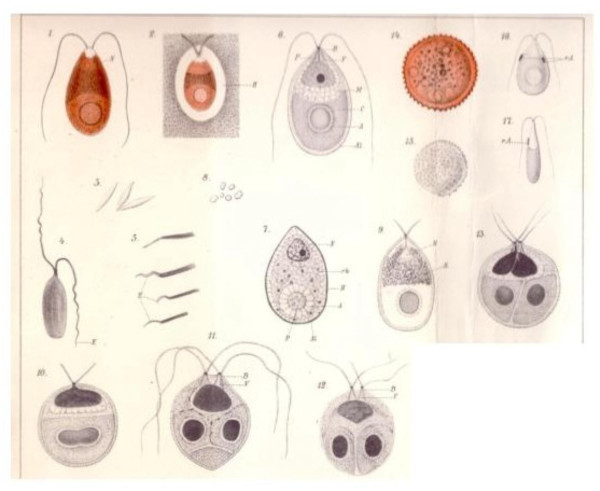
*Dunaliella salina *(1–17) preserved with different fixation techniques; (3), pigment crystals; (8), granules (starch?); (10–13), division stages; (14,15), aplanospores; (16–17), and green cells (*D. viridis*?), as drawn by Hamburger (1905) [7].

Teodoresco studied material collected from a Romanian salt lake, while Hamburger worked with samples sent to her from the salterns of Cagliari, Sardinia. Both authors presented detailed drawings of the organisms (Fig. 2 and 3) and provided extensive information on its morphology, cell structure, reproduction, behavior and ecology. A formal description of the genus *Dunaliella*, named in honor of Dunal who had first seen these organisms in salterns in France almost seventy years earlier, and of the first two species within the genus, *D. salina *and *D. viridis*, was published in 1906 [20].

The papers by Teodoresco and Hamburger were soon followed by others. Noteworthy studies in the early years of *Dunaliella *research are articles by Cavara [21], who extended the study of the organism in the Cagliari, Sardinia salterns, a study of the algae in the Salton Sea, California [22], a series of ecological papers by Labbé based on observations made in the salterns of Le Croisic on the Atlantic coast of France [23-25], articles by Baas Becking and coworkers, who collected specimens from all over the world [26-28], and the taxonomic studies by Hamel [29] and Lerche [30].

## 4. The Taxonomy of the Genus *Dunaliella*

*Dunaliella *is a genus of unicellar algae belonging to the family Polyblepharidaceae. Its cells lack a rigid cell wall, and they reproduce by longitudinal division of the motile cell or by fusion of two motile cells to form a zygote.

Teodoresco [2,20] described two species: *D. salina *and *D. viridis*. *D. salina *has somewhat larger cells, and under suitable conditions it synthesizes massive amounts of carotenoid pigments, coloring the cells brightly red. *D. viridis *never produces such red cells. It is interesting to note that in the early years there have been extensive discussions whether indeed two species are present or whether the red and the green cells represent different forms of the same species. For example, Blanchard [13] and Hamburger [7] considered the green cells as juvenile stages of the red ones. Labbé [23,24] was of the opinion that differences in salt concentration of the environment are responsible for the different colors of the cells. Upon transferring of saltern brine samples to a lower salinity he grew a form of *Dunaliella *adapted to fresh water and lacking the brown-red pigment. His statements that:

"En ce qui concerne les facteurs de la transformation, l'hypothèse simpliste de Teodoresco ne peut être conservée et it ne s'agit pas là de deux espèces distinctes (*D. salina *et *D. viridis*). Il s'agit d'une alternance de formes due aux changements de milieu."

[Concerning the factors of the transformation, the simplistic hypothesis of Teodoresco cannot be maintained, and we do not have here two distinct species (*D. salina *and *D. viridis*). We deal with an alternation of forms due to environmental changes.]

and:

"L'organisme qui colore en rouge les marais salants et à qui nous pouvons conserver le nom de *Dunaliella salina *n'est que la phase ultime de l'évolution d'un flagellé chlorophyllien voisin de Volvocinées, très eurihyalin, qui en eau sursalée donne les formes sténohyalines non réversibles aux formes chlorophylliennes, et colorées par un hématochrome."

[The organism which colors the salterns red and for which we can conserve the name *Dunaliella salina *is nothing but the final phase in the development of a very euryhyaline chlorophyll-containing flagellate related to the Volvocinae, which in hypersaline water produces stenohyaline foms that cannot revert to chlorophyll-containing forms, and are colored by a hematochrome.]

have not withstood the test of time. We now know that not all *Dunaliella *species produce massive amounts of carotene, and those that can, do so only under suitable conditions (exposure to high light intensities, nutrient limitation, etc., see also section 6 below). Lerche [30] thus saw that under suitable conditions all red clones became green, but after several weeks they turned olive to yellow-green and after several months they were red again.

Additional species were later added to the genus, especially thanks to the in-depth studies by Lerche [30] and Butcher [31] (Table 2). Lerche investigated material collected from salt lakes in Romania and in California, as well as the above-mentioned Cagliari salterns. She concluded that the former species *D. viridis *is heterogeneous and should be split into several new species. Thus the species *D. media*, *D. euchlora*, *D. minuta*, and *D. parva *were created. It must be stressed here that not all species mentioned tolerate the extremely high salt concentrations in which *D. salina *and *D. viridis *are found in nature. Some are typically marine organisms that were never reported to occur in hypersaline environments.

**Table 2:** Selected *Dunaliella *species

Name	Author, year
*D. salina*	Teodoresco, 1905, 1906 [2,20]
*D. viridis*	„
*D. peircei*	Nicolai and Baas Becking, 1935 [28]
*D. parva*	Lerche, 1937 [30]
*D. media*	„
*D. euchlora*	„
*D. minuta*	„
*D. tertiolecta*	Butcher, 1959 [31]
*D. primolecta*	„
*D. quartolecta*	„
*D. polymorpha*	„

An in-depth taxonomic treatment of the genus was given in Massyuk's 1973 monograph [32]. She divided the genus into two subgenera, *Dunaliella *(23 species) and *Pascheria *(5 species), the latter consisting of freshwater species only. Some of the species recognized by Massyuk may eventually be found to be polymorphic forms of a single taxon [33].

A species of considerable interest is *Dunaliella acidophila*, isolated from acidic waters and soils in the Czech Republic and in Italy [34,35]. This is not a true halophile but an acidophilic alga that grows optimally at pH values between 0.5 and 2. In recent years it has become a popular research object for the study of adaptation of life to low pH environments [36]. Its taxonomic/phylogenetic affiliation with the halophilic *Dunaliella *species has to my knowledge never been verified.

Molecular phylogeny techniques have been applied to the taxonomic study of *Dunaliella *from 1999 onwards. These studies have encompassed the 18S rRNA genes and the internal transcribed spacer regions, and have been based on gene sequence comparisons as well as on restriction fragment length polymorphism studies. Little correlation was found between the molecular data and the morphological-physiological attributes used in older studies to delineate species within the genus [37,38]. On the basis of 18S rRNA gene sequences, Olmos et al. [39] could differentiate between *D. salina*, *D. parva *and *D. bardawil *as species containing one, two and three introns, respectively, within the 18S rRNA gene. The molecular studies have made it clear that many culture collection strains are probably misnamed, and that some unnecessary species names may have been proposed in the past.

## 5. Life Stages and Sexual Reproduction in *Dunaliella*

**Figure 4:**
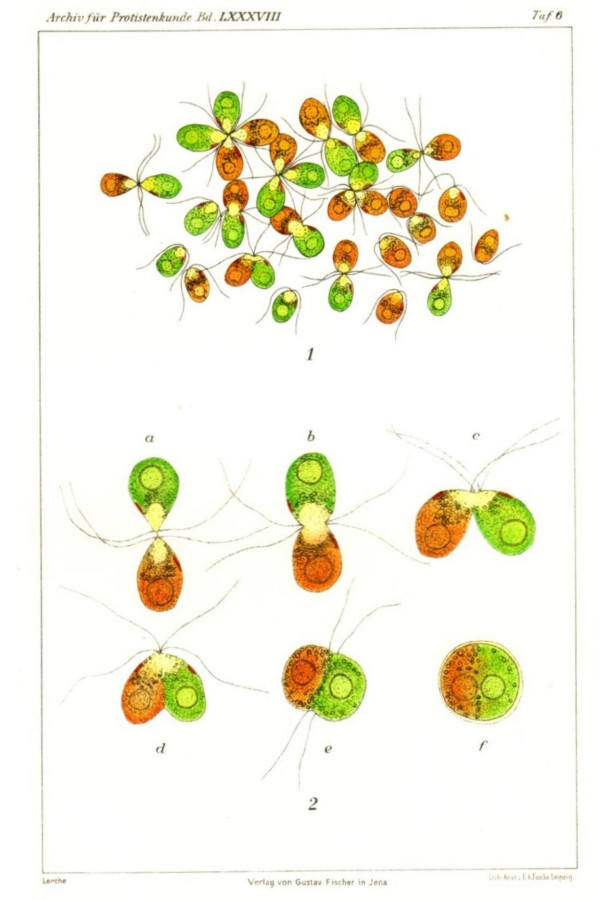
Aggregation of the red and the green form of *Dunaliella salina *(upper part) and zygote formation of *D. salina *(green and red form) (lower part). From [30].

*Dunaliella salina *and some of the other species undergo complex life cycles that encompass, in addition to division of motile vegetative cells, the possibility of sexual reproduction. Fusion of two equally sized gametes to form a zygote was documented in many of the early studies [7,20,29]. We thank a most detailed study of sexual reproduction in *Dunaliella *to Lerche [30], who reported sexual zygote formation in five of the six species studied (*D. salina*, *D. parva*, *D. peircei*, *D. euchlora*, and *D. minuta*). She reported zygote formation in *D. salina *to be induced by a reduction in salt concentration from 10 to 3%. In the process first the flagella touch, and then the gametes form a cytoplasmic bridge and fuse. The zygote has a thick outer layer. It can withstand exposure to freshwater and also survive prolonged periods of dryness. These zygotes germinate with the release of up to 32 haploid daughter cells through a tear in the cell envelope [30]. It is well possible that the cyst-like structures observed by Oren et al. [40] at the end of a bloom of green *Dunaliella *cells in the Dead Sea in 1992 were actually such zygotes. In this case, however, the formation of these rounded, thick-walled cells took place at a time of an increase in water salinity. Lerche [30] performed a series of elegant experiments in which carotenoid-rich red cells were crossed with green cells, enabling the investigator to follow the fusion of the two parent cells to form a zygote. A few of her drawings to illustrate the process are reproduced in Fig. 4. The possibility of formation of asexual resting cysts by *D. salina *was indicated by Hamburger [7], a finding that was disputed by Lerche. However, more recently, Loeblich [41] has reported formation of such cysts in media of reduced salinity (for a discussion see also [42]).

Some *Dunaliella *species can also develop a vegetative palmelloid stage consisting of round non-motile cells. Lerche [30] has documented this phenomenon in *D. salina *cultures at lowered salinities, and Brock [43] observed such palmelloid forms of *Dunaliella *in benthic algal mats of Great Salt Lake, Utah.

## 6. Carotenoid Pigments of *Dunaliella*

The pigment responsible for the brightly red coloration displayed by *D. salina*, often designated in the older literature as "hematochrome", was recognized already very early as a carotenoid. As such it was identified by Blanchard [13], and Teodoresco [20], Lerche [30] and Ruinen [44] confirm this identification based on the solubility of the pigment in alcohol and in ether and on the blue color formed in the presence of concentrated sulfuric acid.

Before the modern electron microscope showed the *β*-carotene as granules between the thylakoids of the cell's single chloroplast, considerable differences of opinion existed regarding the intracellular location of this red carotenoid pigment. Thus, both Teodoresco [2,20] and Labbé [23] stated that the red pigment was distributed all over the cells' cytoplasm. Relating to a different claim by Hamburger [7], Teodoresco [20] wrote:

"je n'hésite pas à croire que ce pigment imprègne tout le corps des zoospores, excepté, bien entendu, l'extrémité antérieure, à l'endroit de l'insertion des flagellums."

[I don't hesitate to believe that the pigment impregnates the whole body of the zoospores, except, of course, the extreme anterior part, on the place where the flagella are inserted.]

Likewise, Hamel [29] claimed that at elevated salt concentrations, *D. salina *forms "hematochrome" that penetrates not only the "chromophore" (= chloroplast) but the entire cytoplasm as well. On the other hand, Hamburger believed the red pigment to be located as small droplets (which is true, see e.g. [45,46]), but she was mistaken about the location of the pigment:

"Er tritt in Form kleiner Tröpfchen auf, und ist, wie mir sicher scheint, nur der äußeren Alveolarschicht des Plasmas eingelagert, während das Chromatophor Träger des grünen Farbstoffes ist. Die Bemerkung Teodoresco's "hématochrome imprégnant non seulement le chromatophore, mais encore tout le corps des individus âgés", stimmt mit meinen Beobachtungen nicht überein."

[It occurs in the form of small droplets, and is, as seems sure to me, only deposited in the outer alveolar layer of the plasma, while the chromatophore is the bearer of the green pigment. The remark by Teodoresco that "the hematochrome that impregnates not only the chromatophore, but also the whole body of adult individuals" does not correspond with my observations.]

Baas Becking [27] correctly located the red-orange pigment in the chloroplast, and Leche [30] realized that the carotene masks the chlorophyll, so that the chloroplast can assume all shades from orange-red to yellow-green, olive and green:

"Der rote Farbstoff ist in Form öliger Tröpfchen zwischen den Wabe des Chloroplasten eingelagert und nicht wie Hamburger (1905) annimmt, in der äußeren Alveolarschicht des Protoplasmas."

[The red pigment is located in the form of oily droplets between the honeycomb structure of the chloroplast and not, as Hamburger (1905) assumes, in the outer cytoplasmic layer of the protoplast.]

*β*-Carotene, the major carotenoid accumulated by *D. salina *and *D. bardawil*, is a valuable chemical, in high demand as a natural food coloring agent, as pro-vitamin A (retinol), as additive to cosmetics, and as a health food [47]. Some *Dunaliella *strains can accumulate very large amounts of this carotenoid. Thus, as much as 13.8% of the total dry organic matter in the *D. salina *community in Pink Lake, Victoria, Australia, was estimated to be *β*-carotene [48]. Also in culture some strains may contain up to 10% and more of *β*-carotene in their dry weight, including a large percentage of the 9-*cis *isomer [46]. Therefore the biotechnological potential of *Dunaliella *as a source of *β*-carotene was investigated already relatively early. The first pilot plant for *Dunaliella *cultivation for *β*-carotene production was established in the USSR in 1966 [49,50]. The commercial cultivation of *Dunaliella *for the production of *β*-carotene throughout the world is now one of the success stories of halophile biotechnology [51-53]. Different technologies are used, from low-tech extensive cultivation in lagoons to intensive cultivation at high cell densities under carefully controlled conditions [54].

One of the methods used in such biotechnological operations to induce massive carotenoid accumulation is reduction of the growth rate by deprivation of nutrients. That a high carotenoid content of the cells may be caused by nutrient limitation as well as by high light intensities was already reported by Lerche [30]:

"Da die Rotfärbung besonders in alten Kulturen auftrat, lag die Annahme nahe, sie in Zusammenhang mit den Ernährungsbedingungen und speziell mit dem Fehlen eines oder mehrerer Stoffe zu bringen. Da Phosphor und Stickstoff bei der pflanzlichen Ernährung häufig die Stoffe sind, die im Minimum vorhanden sind, wurde das Augenmerk zunächst auf diese Stoffe gerichtet."

[As the red coloration occurred especially in old cultures, it was reasonable to assume a correlation with the nutritional conditions and in particular with the lack of one or more compounds. As phosphorus and nitrogen are in plant nutrition often the substances present in limiting amounts, we directed our attention first of all to these substances.]

## 7. Population Dynamics of *Dunaliella *in Salt Lakes and Salterns

Only few studies have been devoted to the quantitative evaluation of *Dunaliella *populations in salt lakes and salterns, the dynamics of their appearance and decline, and their contribution to the primary production in their habitats. Stephens and Gillespie (1976) reported measurements of the primary production in the south arm of Great Salt Lake, Utah, performed in 1973 (salinity around 135 g/l). Post [56] reported that in the cold season, round cyst-like cells of *D. salina *increased in numbers in the Great Salt Lake, especially on the lake's bottom. In the Dead Sea, green *Dunaliella *cells have been reported since the 1940s [57]. The first quantitative estimates of the *Dunaliella *population in the lake were made in 1964, and showed very high numbers: up to 4 × 10^4 ^cells per ml of surface water (sampling season not specified) [58]. Systematic monitoring of the population density at different seasons and depths in the Dead Sea from 1980 onwards have yielded a clear picture of the factors that determine development of the alga in this unusual environment. High concentrations of magnesium and calcium ions are known to be inhibitory to *Dunaliella *since Baas-Becking's earlier studies [27]. *Dunaliella *blooms therefore occur in the Dead Sea only when during unusually wet winters the upper water layers of the lake become sufficiently diluted to enable growth, and when phosphate, the limiting nutrient, is available. Such events have been observed in 1980 and again in 1992 [40,59].

Surprisingly, very little is known about the factors that determine the dynamics of *Dunaliella *in saltern pond systems. It is therefore interesting to note that some of the most in-depth studies on this topic were performed in the early 1920s in the salterns of Le Croisic on the Atlantic coast of France, where salt making is a seasonal operation. Labbé [23,25] showed changes in the algal community structure and related these to changes in salinity ("osmotic pressure; viscosity") of the brine, but he also recognized the role of the light intensity and the water temperature, as well as that of the pH. Based on the faulty assumption that the smaller green and the larger red *Dunaliella *cells are stages in the development of a single organism (see section 4 above), he described an annual cycle in which in the beginning of the winter few red motile cells ("érythrospores") and smaller green motile cells ("chlorospores") are present [24]. Dilution of the water by winter rains triggers the formation of red cysts ("érythrocystes"), but the "chlorospores" develop rapidly, conjugate, and form "chlorocystes". When the salt concentration increases in the summer season, red motile cells start to appear, always accompanied by green cells:

"Peu à peu, les érythrospores provenant de chlorospores prolifèrent, et leur dominance est fonction de la concentration saline."

[Gradually the "erythrospores" that are formed from "chlorospores" proliferate, and their dominance is a function of the salt concentration.]

## 8. Cultivation and Salt Tolerance of *Dunaliella*

**Figure 5:**
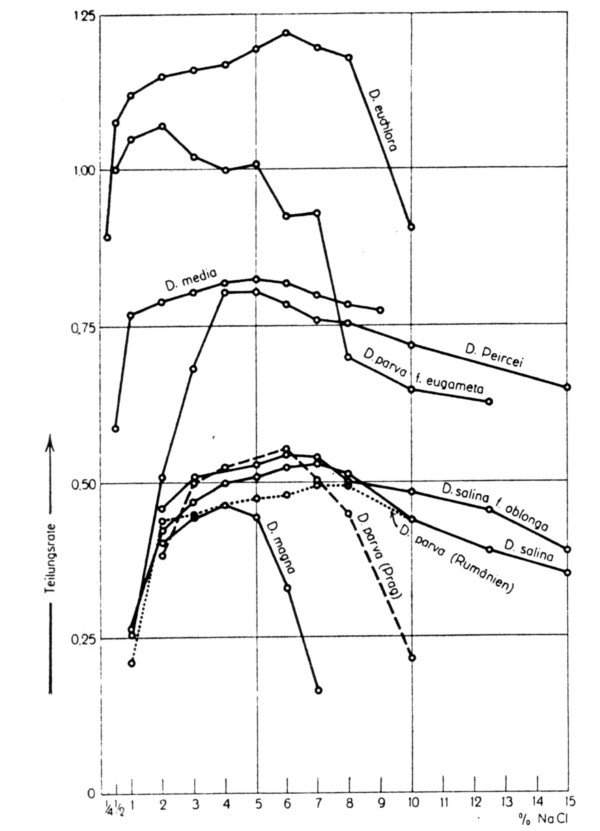
Division rate ("Teilungsrate") (as number of divisions per day) of different *Dunaliella *isolates belonging to several species, as a function of the NaCl concentration of the medium. From [30].

The first controlled experiments to evaluate the effect of salinity on the growth rate of different *Dunaliella *isolates were reported in the 1930s. Baas-Becking [27] observed that *D. viridis *thrives equally well over the whole range of 1–4 M (6–23%) NaCl and over the pH range 6–9. He found calcium and magnesium ions in high concentrations to be inhibitory. More detailed and well-documented experiments, using a variety of species and isolates, were reported by Lerche [30]. She found most isolates to grow optimally between 2 and 8% salt, with very slow growth, if at all, at salt concentrations above 15% (Fig. 5). Between 0.47 and 1.22 divisions per day were recorded under optimal conditions.

The nutritional requirements of different *Dunaliella *strains were investigated in-depth by Gibor [60], Johnson et al. [61], Van Auken and McNulty [62], and others, enabling the optimization of media to grow the alga. Optimal salt concentrations for cultivation varies according to the strain, with values reported for *D. viridis *around 6%, for *D. salina *around 12% [42], while different Great Salt Lake isolates had optima of 10–15% or even 19% salt [43,62]. A general trend, observed in all these studies, is that the actual salinity of the environments from which the strains had been isolated was always much higher than the salt concentration found to be optimal in laboratory experiments. This may well reflect the fact that growth of an organism occurs in a certain environment not necessarily means that that environment is optimal for its development, but rather that the organism performs there better than all its competitors.

## 9. Osmotic Behavior of *Dunaliella *Cells

*Dunaliella *cells lack a rigid cell wall, and the cell is enclosed solely by a thin elastic plasma membrane. As a result, the cells' morphology is strongly influenced by osmotic changes. This was documented already in the early days. The descriptions by Teodoresco [2] are very exact here, and they deserve to be cited unabridged:

"Ces zoospores sont dépourvues de membrane cellulosique; celle-ci est représentée par une enveloppe qui possède une certaine souplesse et une certaine extensibilité, qui permet au corps de prendre les formes assez variées, suivant la concentration de l'eau. A ce point de vue, le genre *Dunaliella *diffère totalement de toutes les espèces de *Chlamydomonas *..."

[These zoospores are devoid of a cellulose cell wall; instead there is a cell envelope that possesses a certain flexibility and a certain elasticity, which allows the body to take quite different forms, in accordance with the [salt] concentration of the water. In this respect the genus *Dunaliella *differs completely from all species of *Chlamydomonas *...]

and:

"Ainsi, si nous plaçons une goutte d'eau salée, contenant des zoospores, sur le porte-objet, on constate, au microscope, qu'elles se présentent sous la forme mentionée plus haut. Mais si nous laissons la goutte s'évaporer un peu, on observe que le corps commence à s'allonger et à se difformer ... ; si alors nous ajoutons à la préparation une goutte d'eau douce, les zoospores s'arrondissent brusquement .... Cette expérience, que j'ai répétée un trés grand nombre de fois, m'a toujours donné les mêmes résultats."

[Thus, when we place a drop of salt water that contains zoospores [= motile vegetative cells] on a microscope slide, one detects in the microscope that these present themselves in the above-described form. However, when we let the drop evaporate a little, one observes that the body starts to elongate and to lose its shape. ... ; when we then add to the preparation a drop of fresh water, the zoospores suddenly round up. .... This experiment, which I have repeated a great number of times, has always given me the same results.]

The phenomena described above are illustrated in Fig. 2, drawings 9–29 and 30–31, respectively. Teodoresco further writes:

"Si à une goutte d'eau salée on ajoute une goutte plus grande d'eau douce, ce qui amène une abaissement brusque de la concentration, les zoospores non seulement s'arrondissent, mais encore cessent leurs mouvements; le volume du corps augmente et devient parfois deux fois plus grand et à la fin la zoospore éclate. La cause de cet éclatement n'est pas difficile à comprendre: c'est l'action méchanique de la pression osmotique trop élevée par rapport à la densité diminuée du milieu ambiant."

[If to a drop of salt water one adds a larger drop of fresh water, which leads to a sudden drop in concentration, the zoospores not only round up, but in addition cease their movements; the volume of the body increases and sometimes becomes twice as large, and finally the zoospore bursts. The cause of this burst is not difficult to understand: it is the mechanical action of the too high osmotic pressure in comparison to the decreased density of the ambient medium.]

Lerche [30] likewise observed the osmotic changes that occur when the salt concentration is changed. She noted that when a drop of *D. salina *cells suspended in 20% salt is flooded with distilled water, a large fraction of the cells burst, but some cells survived the treatment.

## 10. Intracellular Salt and Solute Concentrations of *Dunaliella*

Marrè and Servetta ([63], as cited in [61]) described measurements of the freezing point of the cytoplasmic fluid of *D. salina *to obtain information on the intracellular salt concentration. The results indicated an apparent "salt" concentration that exceeded the 3.9 M salt in which the cells were grown. At the time it was postulated that NaCl is taken up through the allegedly very permeable cell membrane during salt upshock, followed by free water flux to equalize intracellular and extracellular osmotic pressures [63-65].

That the salt concentrations within *Dunaliella *cells cannot be that high, was convincingly shown by the enzymological studies by Johnson et al. (1968), who demonstrated that some of the key enzymes of the algal metabolism such as pentose phosphate isomerase, ribulose bisphosphate carboxylase, glucose-6-phosphate dehydrogenase and phosphohexose isomerase, are strongly inhibited by NaCl. We now know that the intracellular ionic concentrations of *Dunaliella *are very low indeed. Using lithium ions as a marker for the extracellular water space to estimate the intracellular volume, the intracellular Na^+ ^concentrations, both in cells grown in 0.5 M and in 4 M NaCl, was found not to exceed 100 mM [66]. Such low intracellular Na^+ ^levels are achieved by the activity of a Na^+^/H^+ ^antiporter in the cytoplasmic membrane [67], as well as by direct electron transport-coupled Na^+ ^extrusion [68].

The enigma of the apparent incompatibility between the low intracellular ionic concentrations and the need for osmotic equilibrium of the cells' contents with the external medium was solved with the discovery that the cells accumulate photosynthetically produced glycerol as osmotic, "compatible" solute. It is interesting to note that the first experiments in which the effects of glycerol on *Dunaliella *were tested had already been performed by Teodoresco [20], almost hundred years ago. He examined the effect of glycerol and other non-ionic compounds that normally cause plasmolysis. He observed that *D. salina *cells temporarily lose their motility when suspended in 50% glycerol, but that motility is rapidly restored when the glycerol concentration is then slightly lowered in a humid environment. With 75% glycerol results were largely similar, except that a large fraction of cells died, and in 100% glycerol only few cells survived.

The first indications that glycerol is accumulated by *Dunaliella *to provide osmotic balance can be found in a short paper published in 1964 by Craigie and McLachlan [69]. They incubated *D. tertiolecta *with ^14^CO_2_, then extracted the cells with ethanol, separated the neutral fraction containing soluble carbohydrates and related compounds using ion exchange procedures, and characterized the compounds by two-dimensional paper chromatography and autoradiography. When the salinity of the medium was increased 100-fold from 0.025 to 2.5 M, 94-fold more radioactivity was found in the neutral fraction. Glycerol amounted to 56, 76, and 81% of the radioactivity of the neutral fraction extracted from cells incubated in 0.025, 0.5, and 2.5 M NaCl, respectively, most of the remainder probably consisting of soluble polysaccharides. In a subsequent study, Wegmann [70] confirmed that the proportion of the radiolabel from ^14^C-bicarbonate that ends up as glycerol increases with increasing salt concentration up to 2.8 M. He postulated that "The glycerol formation is considered to be a protective mechanism for the survival of *Dunaliella *in its natural habitat".

The role that glycerol plays in the salt adaptation of *Dunaliella *was firmly established by the studies of Ben-Amotz and Avron [71] and Borowitzka and Brown [72]. The concept of "compatible solutes", a term coined by Duncan Brown to indicate solutes that not only contribute to the osmotic status of the cell but also maintain enzyme activity under conditions of low water activity, was largely based on the study of the function of glycerol in *Dunaliella*.

Intracellular glycerol concentrations in *Dunaliella *can be very high indeed: cells grown in 4 M NaCl were reported to contain approximately 7.8 M glycerol inside, equivalent to a solution of 718 g l^-1 ^glycerol in water [73]. Maintenance of such a high concentration requires special properties of the cell membrane in view of the fact that most biological membranes are relatively permeable to glycerol. It has been established that *Dunaliella *possesses a membrane with an unusually low permeability for glycerol [74,75], and this enables the cells to keep the glycerol inside the cell. The causes of the low glycerol permeability of the *Dunaliella *membrane are still not fully understood.

Attempts have been made to exploit the high concentrations of glycerol accumulated by *Dunaliella *as the basis for the commercial production of this compound. Although technically the production of glycerol from *Dunaliella *was shown to be possible [51,52,76] economic feasibility is low, and to my knowledge no biotechnological operation presently exists that exploits the alga for glycerol production.

## 11. Proteomics Approaches to the Understanding of Salt Tolerance in *Dunaliella*

A versatile organism such as *Dunaliella *that can adapt to a wide variety of salt concentrations can be used as a convenient model to study the formation of specific proteins as a function of changes in medium salinity. Such proteomic approaches have led to some interesting observations in recent years.

A number of such studies were directed to the detection of changes in the protein content of the cytoplasmic membrane, whose outer side is exposed to the medium salinity, when the cells are shifted from low to high salinity. Two membrane proteins were strongly induced by salt upshock, one with an apparent molecular mass of 60 kDa [77] and one of 150 kDa [78]. These proteins have been purified and characterized. The 60 kDa protein is a carbonic anhydrase that apparently helps the cell to take up carbon dioxide in concentrated brines in which the solubility of gases is decreased [79]. The 150 kDa protein is an unusual transferrin-like protein, involved in the transport of iron into the cell [80].

With a study published in 2004 by Liska et al. [81], *Dunaliella *research has entered the era of modern proteomics. Comparison of protein patterns of low-and of high-salt-grown cells were compared on two-dimensional gels led to the identification of 76 salt-induced proteins. Among the proteins up-regulated following salinity stress were key enzymes in the Calvin cycle, enzymes involved in starch mobilization and in redox energy production, regulatory factors in protein biosynthesis and degradation, and a homolog of bacterial Na^+^-redox transporters. The results indicate that *Dunaliella *responds to transfer to a high salinity by enhancement of photosynthetic CO_2 _assimilation and by diversion of carbon and energy resources for synthesis of glycerol. This beautiful study is a worthy conclusion of the first century of *Dunaliella *research, and provides us with a preview of the kind of information that may be expected to be obtained in the coming years, using approaches of genomics, proteomics and systems biology.
